# Velvet antler water extract protects porcine oocytes from lipopolysaccharide‐induced meiotic defects

**DOI:** 10.1111/cpr.13392

**Published:** 2023-01-03

**Authors:** Jingyue Chen, Rui Wang, Chunxiao Liu, Bo Xiong, Yilong Miao, Cong Rao, Huimin Sun, Qian Gao, Baozeng Xu

**Affiliations:** ^1^ Institute of Special Animal and Plant Sciences, Chinese Academy of Agricultural Sciences Changchun China; ^2^ College of Animal Science and Technology Nanjing Agricultural University Nanjing China; ^3^ College of Plant Protection Nanjing Agricultural University Nanjing China; ^4^ College of Veterinary Medicine Nanjing Agricultural University Nanjing China

## Abstract

Previous studies have demonstrated that lipopolysaccharide (LPS), as a central toxic factor of gram‐negative bacteria, can induce oxidative stress and cellular inflammation to result in the impairment of female fertility in different organisms. Particularly, it has harmful effects on the oocyte quality and subsequent embryonic development. However, the approach concerning how to prevent oocytes from LPS‐induced deterioration still remains largely unexplored. We assessed the effective influences of velvet antler water extract (VAWE) by immunostaining and fluorescence intensity quantification on the meiotic maturation, mitochondrial function and sperm binding ability of oocytes under oxidative stress. Here, we report that VAWE treatment restores the quality of porcine oocytes exposed to LPS. Specifically, LPS exposure contributed to the failed oocyte maturation, reduced sperm binding ability and fertilization capability by disturbing the dynamics and arrangement of meiotic apparatuses and organelles, including spindle assembly, chromosome alignment, actin polymerization, mitochondrial dynamics and cortical granule distribution, the indicators of oocyte nuclear and cytoplasmic maturation. Notably, VAWE treatment recovered these meiotic defects by removing the LPS‐induced excessive ROS and thus inhibiting the apoptosis. Collectively, our study illustrates that VAWE treatment is a feasible strategy to improve the oocyte quality deteriorated by the LPS‐induced oxidative stress.

## Graphical abstract

VAWE restores the quality of LPS‐exposed oocytes by enhancing the mitochondrial function and suppressing the apoptosis.

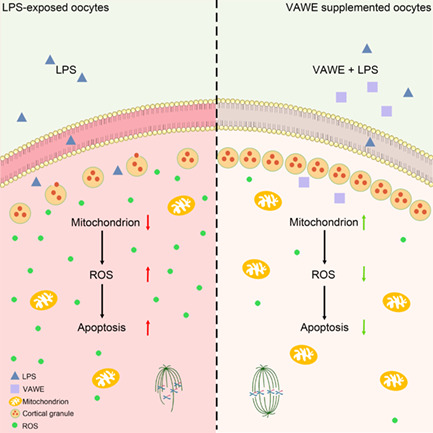

## 1 INTRODUCTION

Gram‐negative bacteria possess an outer protective cell wall that harbours several pathogen‐associated molecular patterns (PAMPs) to trigger the inflammation and cause other toxic effects when they infect a host or are lysed by antibiotics.^1^ Lipopolysaccharide (LPS), as a core component of the outer membrane of gram‐negative bacteria, exists extensively in numerous industrial and external environments, including livestock farms, lumbermills and cotton mills.^2^, ^3^ LPS is a prototypical PAMP which is identified by toll‐like receptor 4 (TLR‐4) protein in complex with CD14 and soluble myeloid differentiation factor 2 (MD2) on the cell surface.^4^, ^5^ After LPS binds to its receptor, inflammatory molecules such as chemokines IL‐8, TNF‐α, IL‐6 and cytokines IL‐1β are released.^4^ The LPS‐induced molecules cause severe pathology by exerting profound regulatory effects on the cellular functions.^6^ In addition, many studies have validated the adverse influences of LPS on the folliculogenesis, oocyte development, ovulation, luteal function, ovarian steroidogenesis, estrus behaviour, and puberty onset in female animals.^7^ Notably, a recent study has found that exposure to LPS destructed the oocyte meiotic maturation due to the reduced m6A levels in pigs.^8^ In general, LPS exposure seriously damages the physiological health and female reproductive performance of animals.^8^, ^9^, ^10^

Velvet antler (VA), a tissue that is separated from elk or deer, is one of the most famous animal‐derived medicine materials.^11^, ^12^, ^13^ As a invigorator of yang and qi acting through the kidney meridian, VA has been widely used in the traditional Chinese medicine to reinforce virility, replenish the crucial essence, nourish the blood, strengthen bones, and accelerate male and female sexual functions.^14^, ^15^, ^16^ Previous reports have indicated that VA had anti‐inflammatory effects on human rheumatoid arthritis fibroblast‐like synoviocytes and alleviated the Parkinson's disease by inhibiting oxidative stress in mice,^17^, ^18^ further demonstrating that VA had many beneficial components and pharmacological actions.^19^, ^20^, ^21^, ^22^ Also, other studies have shown that VA could rescue the sarcoplasmic reticulum Ca^2+^‐ATPase activity and thus enhanced the cardiac function in rats with heart failure.^23^, ^24^ However, whether VA could improve the quality of oocytes under oxidative stress from diverse environmental factors is largely elusive.

In the present study, we examined the potential impacts of velvet antler water extract (VAWE) on the quality of porcine oocytes exposed to LPS. We evaluated the maturation competency and fertilization ability of oocytes. We also assessed the cytoskeleton organization, mitochondrial integrity, and cortical granule dynamics during nuclear and cytoplasmic maturation of oocytes.

## 2 MATERIALS AND METHODS

### 2.1 LPS and VAME treatment

LPS was purchased from Sigma Aldrich (St Louis, MO). VAWE was provided by Dr. Guangyu Li's lab at the Institute of Special Animal and Plant Sciences, Chinese Academy of Agricultural Sciences, and prepared from the same velvet antler in one time. LPS was dissolved in the sterilized water and diluted to a final concentration of 25 μg/ml with maturation medium. VAWE was dissolved in PBS and diluted to working concentrations of 25, 50, 100, 200, 500 μg/ml with maturation medium.

### 2.2 Porcine oocyte collection

Porcine ovaries were obtained from a local abattoir. Within 2 h after slaughtering, abattoir‐derived porcine ovaries were collected and then transported to the laboratory in a physiological saline (0.9% NaCl) containing 500 IU/ml of streptomycin sulphate and penicillin G. The cumulus‐oocyte complexes (COCs) were aspirated with 10 ml disposable syringes from standard (3–6 mm in diameter) follicles. COCs which had compact cumulus cells were transferred to maturation medium for in vitro maturation (IVM). Maturation medium is composed of TCM‐199 (ThermoFisher Scientific, Waltham, MA) supplemented with 0.6 mM cysteine, 10% porcine follicular fluid, 0.2 mM pyruvate, 5 μg/ml insulin, 10 IU/ml of eCG and hCG, 25 μg/ml kanamycin and 10 ng/ml EGF. Twenty to forty germinal vesicle (GV) COCs were grown at 38.5°C and 5% CO_2_ for 26–28 h to metaphase I (M I) stage, and for 44–48 h to metaphase II (MII) stage in a drop of 50–100 μl maturation medium covered with mineral oil.

### 2.3 Fluorescence staining and laser confocal microscopy

Oocytes without cumulus cells were fixed at room temperature (RT) in 4% paraformaldehyde/PBS for 30 min, permeabilized in 1% Triton X‐100/PBS for 8 h, and blocked in 1% BSA/PBS for 1 h. Then denuded oocytes were incubated with γH2AX antibody (Cell Signaling Technology, Danvers, MA; 1:100), LCA‐FITC (Sigma‐Aldrich; 1:100), α‐tubulin‐FITC antibody (Sigma‐Aldrich; 1:200), phalloidin‐TRITC (Sigma‐Aldrich; 1:100) or anti‐human ovastacin antibody (Jurrien Dean lab, NIH; 1:100) at 4°C overnight. Oocytes were next washed in PBST, followed by incubation with the secondary antibodies for 1 h and 10 μg/ml propidium iodide (PI) for 10 min at RT. Lastly, oocytes were mounted on the glass slides for imaging using laser confocal microscope (LSM 900, Carl Zeiss, Germany).

For live staining, oocytes were incubated with MitoTracker Red CMXRos (ThermoFisher Scientific; 1:2000), MitoProbe JC‐1 (ThermoFisher Scientific; 1:100), Dichlorofluorescein Diacetate (Beyotime, Huangzhou, China; 1:800) or Annexin‐V‐FITC (Beyotime, Huangzhou, China; 1:10) at 38.5°C for 30 min, followed by imaging using laser confocal microscope (LSM 900, Carl Zeiss, Germany).

### 2.4 Sperm‐oocyte binding and in vitro fertilization

For sperm‐oocyte binding, porcine sperm were capacitated in the fertilization medium mTBM (modified Tris‐Buffered Medium; 10 mM NaCl, 7.5 mM CaCl_2_·2H_2_O, 3 mM KCl, 11 mM Glucose, 20 mM Tris, 5 mM Na‐pyruvate) at a concentration of 1 × 10^6^ cells/ml at 38.5°C for 1 h. Then, 50 μl sperm was added to the fertilization droplets containing 10–15 matured oocytes for 2 h of incubation, followed by the wash of excessive sperm attached to the oocytes and sperm head staining with Hoechst 33342. For in vitro fertilization (IVF), the incubation of sperm and oocytes was extended to 6–8 h. Fertilized oocytes were further cultured in the porcine zygote medium PZM3 at 38.5°C in an atmosphere of 5% CO_2_.

### 2.5 Statistical analysis

All percentages or values from at least three repeated experiments were expressed as mean ± SEM or SD, and the number of oocytes was labelled in parentheses as (*n*). Data were analysed by unpaired‐samples *t*‐test, provided by GraphPad Prism 7 statistical software. The level of significance was accepted as *P* < 0.05.

## 3 RESULTS

### 3.1 VAWE promotes the in vitro maturation of porcine oocytes exposed to LPS

**FIGURE 1:**
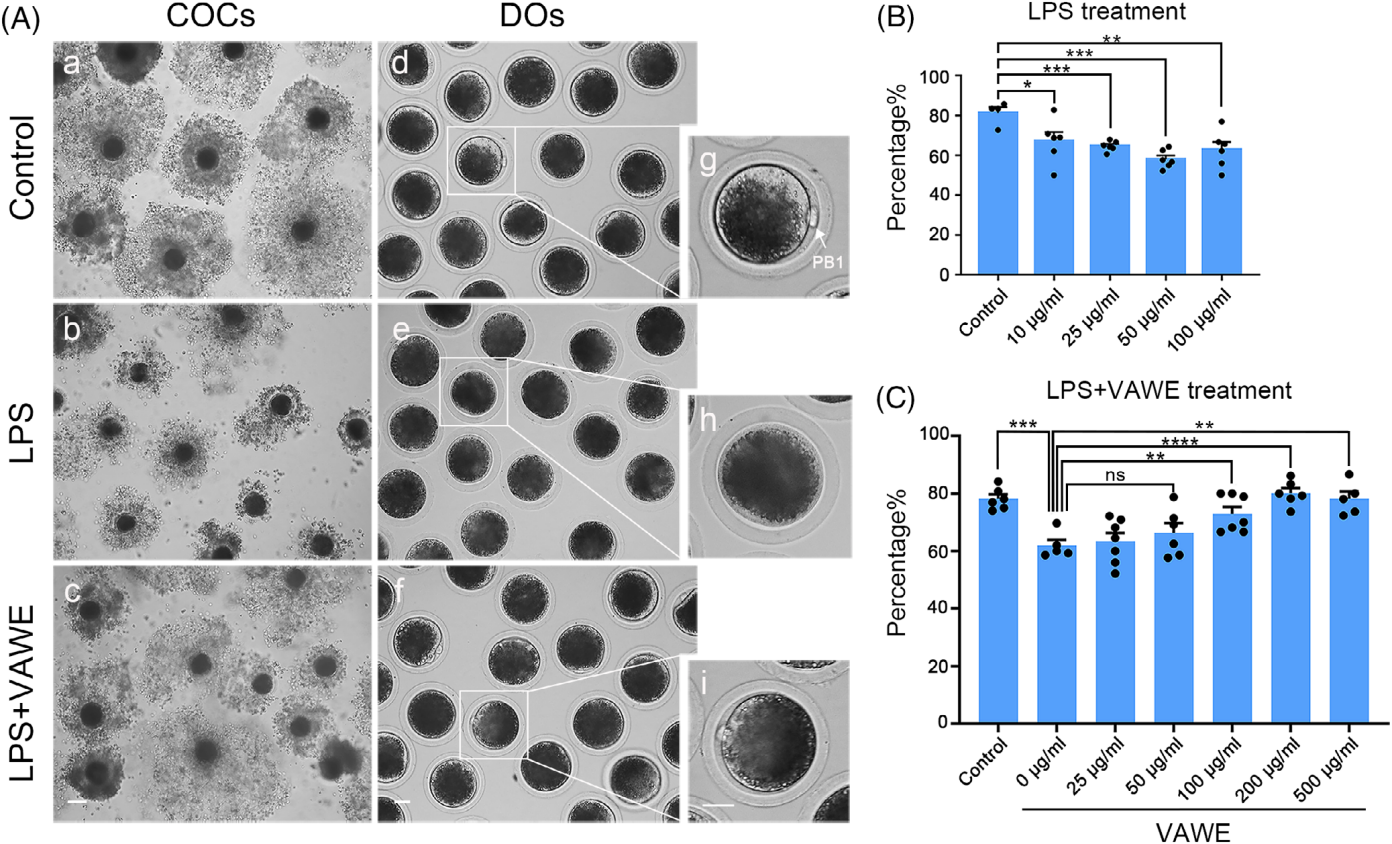
Effects of VAWE supplementation on the in vitro maturation of LPS‐exposed porcine oocytes. (A) Representative images of in vitro matured oocytes in control, LPS‐exposed and VAWE‐supplemented oocytes. Cumulus cell expansion of COCs and PBE of DOs were imaged by the confocal microscope. Scale bars, 120 μm (A–C); 30 μm (d‐f); 30 μm (G–I). (B) The rates of PBE were calculated in LPS‐exposed oocytes at different concentrations (0, 10, 25, 50, 100 μg/ml) after in vitro culture for 44–48 h. (C) The rates of PBE were calculated in VAWE‐supplemented oocytes at different concentrations (0, 25, 50, 100, 200, 500 μg/ml) after in vitro culture for 44–48 h. Data in (B) and (C) were presented as mean percentage (mean ± SEM) of at least three independent experiments. *P < 0.05; **P < 0.01; ***P < 0.001; ****P < 0.0001; ns, no significance

During in vitro maturation (IVM), we added different doses of LPS (10, 25, 50, or 100 μg/ml) in the culture medium to study its effects on the porcine oocyte meiotic progression. It was shown that a large number of cumulus cells were fully expanded around the oocytes in the control group, but became less in the LPS‐exposed group (Figure 1A). We further calculated the frequency of first polar body extrusion (PBE), and observed that exposure to different concentrations of LPS remarkably reduced the PBE rate in varying degrees (control: 81.3 ± 2.9%, *n* = 104; 10 μg/ml LPS: 67.2 ± 4.4%, *n* = 171, *P* < 0.05; 25 μg/ml LPS: 64.7 ± 1.0%, *n* = 151, *p* < 0.001; 50 μg/ml LPS: 58.1 ± 1.9%, *n* = 171, *P* < 0.001; 100 μg/ml LPS: 56.1 ± 3.5%, *n* = 156, *P* < 0.01; Figure 1B). For subsequent studies, 25 μg/ml LPS was chosen because it permitted a certain proportion of oocytes to reach the M II stage.

To determine whether VAWE could mitigate the oocyte meiotic arrest caused by LPS, we supplemented different concentrations of VAWE to IVM culture medium containing 25 μg/ml LPS. Our results displayed that 200 μg/ml VAWE substantially advanced the rate of PBE of LPS‐exposed oocytes and ameliorated the expansion of cumulus cells surrounding COCs (control: 78.2 ± 1.5%, *n* = 331; 0 μg/ml VAWE: 61.9 ± 2.0%, *n* = 187, *p* < 0.001; 25 μg/ml VAWE: 63.4 ± 2.9%, *n* = 208; 50 μg/ml VAWE: 66.4 ± 3.4%, *n* = 226; 100 μg/ml VAWE: 72.9 ± 2.4%, *n* = 194, *P* < 0.01; 200 μg/ml VAWE: 80.1 ± 1.8%, *n* = 248, *P* < 0.0001; 500 μg/ml VAWE: 78.2 ± 2.5%, *n* = 213, *P* < 0.01; Figure 1A,C), suggesting that VAWE supplementation is able to protect oocytes from meiotic failure induced by LPS exposure.

### 3.2 VAWE repairs spindle/chromosome defects in porcine oocytes exposed to LPS

**FIGURE 2:**
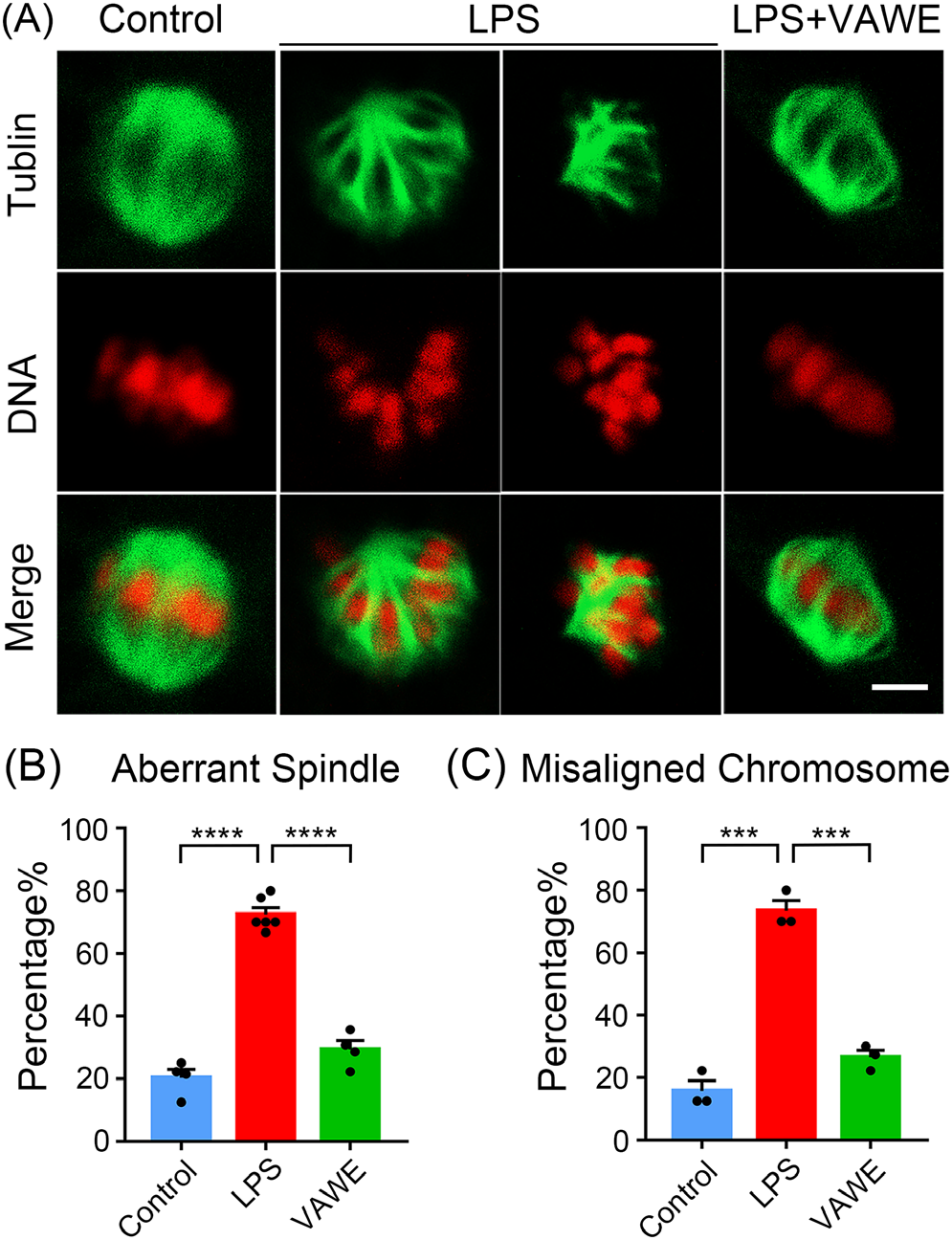
Effects of VAWE supplementation on the spindle/chromosome structure in LPS‐exposed porcine oocytes. (A) Metaphase I oocytes were immunostained with anti‐α‐tubulin‐FITC antibody to represent the spindle morphology and counterstained with propidium iodide (PI) to exhibit the chromosome alignment. Scale bar, 5 μm. (B) The percentage of aberrant spindles was recorded in control, LPS‐exposed and VAWE‐supplemented oocytes. (C) The percentage of misaligned chromosomes was recorded in control, LPS‐exposed and VAWE‐supplemented oocytes. Data in (B) and (C) were expressed as mean percentage (mean ± SEM) of at least three independent experiments. ***P < 0.001; ****P < 0.0001

Considering that nuclear maturation of oocytes requires the proper cytoskeletal assembly, we next investigated whether VAWE had a beneficial impact on the spindle morphologies and chromosome alignment in LPS‐exposed oocytes. By fluorescence staining with α‐tubulin antibody and PI, spindle organization and chromosome alignment were observed in oocytes. As shown in Figure 2A, in the control group, spindles exhibited a normally organized shape and chromosomes were orderly arranged at the equatorial plate, whereas LPS‐exposed group had a higher frequency to display the disorganized spindle morphologies with misalignment of chromosomes (spindle: 20.3 ± 2.7%, *n* = 39 VS 72.4 ± 2.1%, *n* = 58, *P* < 0.0001; chromosome: 15.7 ± 3.2%, *n* = 25 VS 73.3 ± 3.3%, *n* = 30, *P* < 0.001; Figure 2B,C). After supplementation with VAWE, there was a remarkable reduction in the proportion of abnormal spindles and misaligned chromosomes in LPS‐exposed oocytes (spindle: 72.4 ± 2.1%, *n* = 58 VS 29.3 ± 2.8%, *n* = 50, *P* < 0.0001; chromosome: 73.3 ± 3.3%, *n* = 30 VS 26.5 ± 2.3%, *n* = 30, *P* < 0.001; Figure 2B,C), indicating that VAWE restores the deficient structure of spindle and chromosome apparatus in porcine oocytes exposed to LPS.

### 3.3 VAWE maintains the actin polymerization in LPS‐exposed oocytes

**FIGURE 3:**
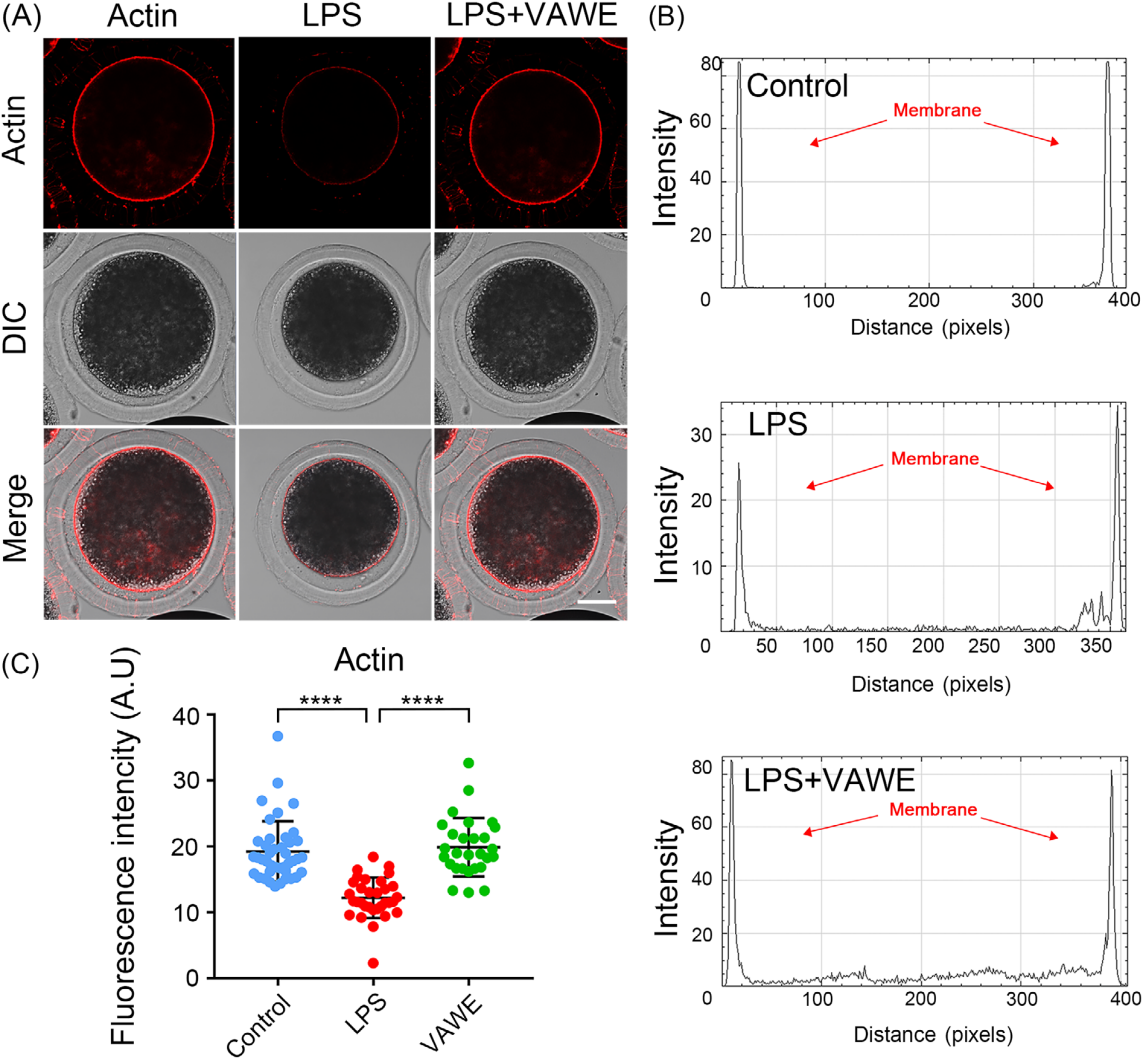
Effects of VAWE supplementation on the actin polymerization in LPS‐exposed porcine oocytes. (A) Representative images of actin filaments in control, LPS‐exposed and VAWE‐supplemented oocytes. Scale bar, 30 μm. (B) The graphs showed the fluorescence intensity profiling of actin filaments in control, LPS‐exposed and VAWE‐supplemented oocytes. Lines were drawn through the oocytes, and pixel intensities were quantified along the lines. (C) The fluorescence intensity of actin signals was quantified in control, LPS‐exposed and VAWE‐supplemented oocytes. Data in (C) were shown as mean value (mean ± SD) of at least three independent experiments. ****P < 0.0001

On account of the fact that microfilament takes a vital part in the meiotic cell polarization and spindle positioning, we used phalloidin to display the polymerization of actin filaments. According to the results from fluorescent images, a uniform distribution of actin filaments was observed on the plasma membrane with strong signals in the control group (Figure 3A). By comparison, porcine oocytes exposed to LPS displayed less actin signals, as indicated along the line drawn through the oocyte by fluorescence profiling and quantification (19.2 ± 0.7%, *n* = 41 VS 12.2 ± 0.5%, *n* = 32, *P* < 0.0001; Figure 3A–C). Strikingly, VAWE supplementation increased the actin signals on the plasma membrane in LPS‐exposed oocytes as assessed by the measurement of fluorescence intensity (12.2 ± 0.5%, *n* = 32 VS 19.9 ± 0.8%, *n* = 28, *P* < 0.0001; Figure 3C). To Sum up, these data imply that the recovery of VAWE on the oocyte meiotic defects induced by LPS involves in the actin dynamics.

### 3.4 VAWE restores the distribution and function of mitochondria in porcine oocytes exposed to LPS

**FIGURE 4:**
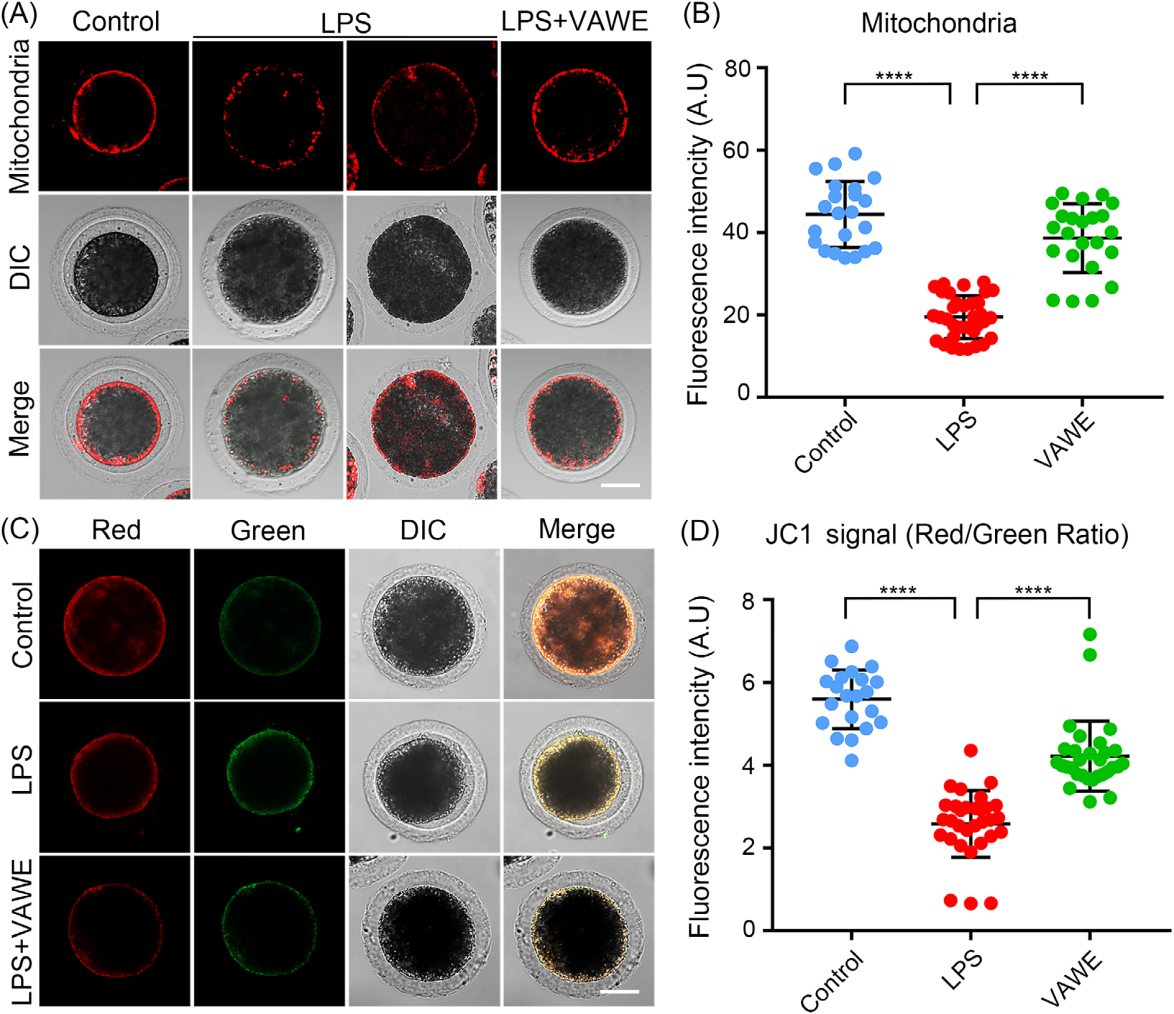
Effects of VAWE supplementation on the distribution and function of mitochondria in LPS‐exposed porcine oocytes. (A) Representative images of mitochondrial distribution in control, LPS‐exposed and VAWE‐supplemented oocytes. Scale bar, 40 μm. (B) The fluorescence intensity of mitochondrial signals was measured in control, LPS‐exposed and VAWE‐supplemented oocytes. (C) Mitochondrial membrane potential (ΔΨm) was tested by JC‐1 staining in control, LPS‐exposed and VAWE‐supplemented oocytes (Red, high ΔΨm; Green, low ΔΨm). Scale bar, 40 μm. (D) The ratio of red to green fluorescence intensity was calculated in control, LPS‐exposed and VAWE‐supplemented oocytes. Data in (B) and (D) were presented as mean values (mean ± SD) of at least three independent experiments. ****P < 0.0001

Given that mitochondria provide the energy for many biological processes including cytoskeleton assembly during oocyte maturation, its distribution has been considered as one of the major indexes for the oocyte cytoplasmic maturation. In the control group, we found that most of mitochondria in the oocytes aggregated in the subcortical region around lipid droplets, whereas this particular localization pattern disappeared in oocytes exposed to LPS (Figure 4A). Based on the quantitative fluorescence intensity analysis, we validated that signals of mitochondria in LPS‐exposed oocytes prominently declined compared to the control oocytes (44.4 ± 1.7%, *n* = 22 VS 19.4 ± 0.9%, *n* = 34, *P* < 0.0001), which was prevented by the treatment with VAWE (19.4 ± 0.9%, *n* = 34 VS 38.6 ± 1.7%, *n* = 23, *P* < 0.0001; Figure 4A,B). Moreover, mitochondrial membrane potential (ΔΨm) was detected by JC‐1 staining to assess the mitochondrial function. When mitochondria had a high membrane potential, they fluoresced in red to represent the aggregates of JC‐1, and mitochondria with a low membrane potential fluoresced in green to show the monomers of JC‐1 (Figure 4C). As measured by fluorescence intensity, the ratio of red to green fluorescence in LPS‐exposed oocytes was dramatically lower than that in control oocytes, but elevated after VAWE supplementation (5.6 ± 0.2%, *n* = 21, *P* < 0.0001 VS 2.6 ± 0.1%, *n* = 31 VS 4.2 ± 0.2%, *n* = 30, *P* < 0.0001; Figure 4C,D). In brief, these results indicate that VAWE recovers the impairment of mitochondria in oocytes exposed to LPS.

### 3.5 VAWE rescues the abnormal distribution of cortical granules and ovastacin in LPS‐exposed oocytes

**FIGURE 5:**
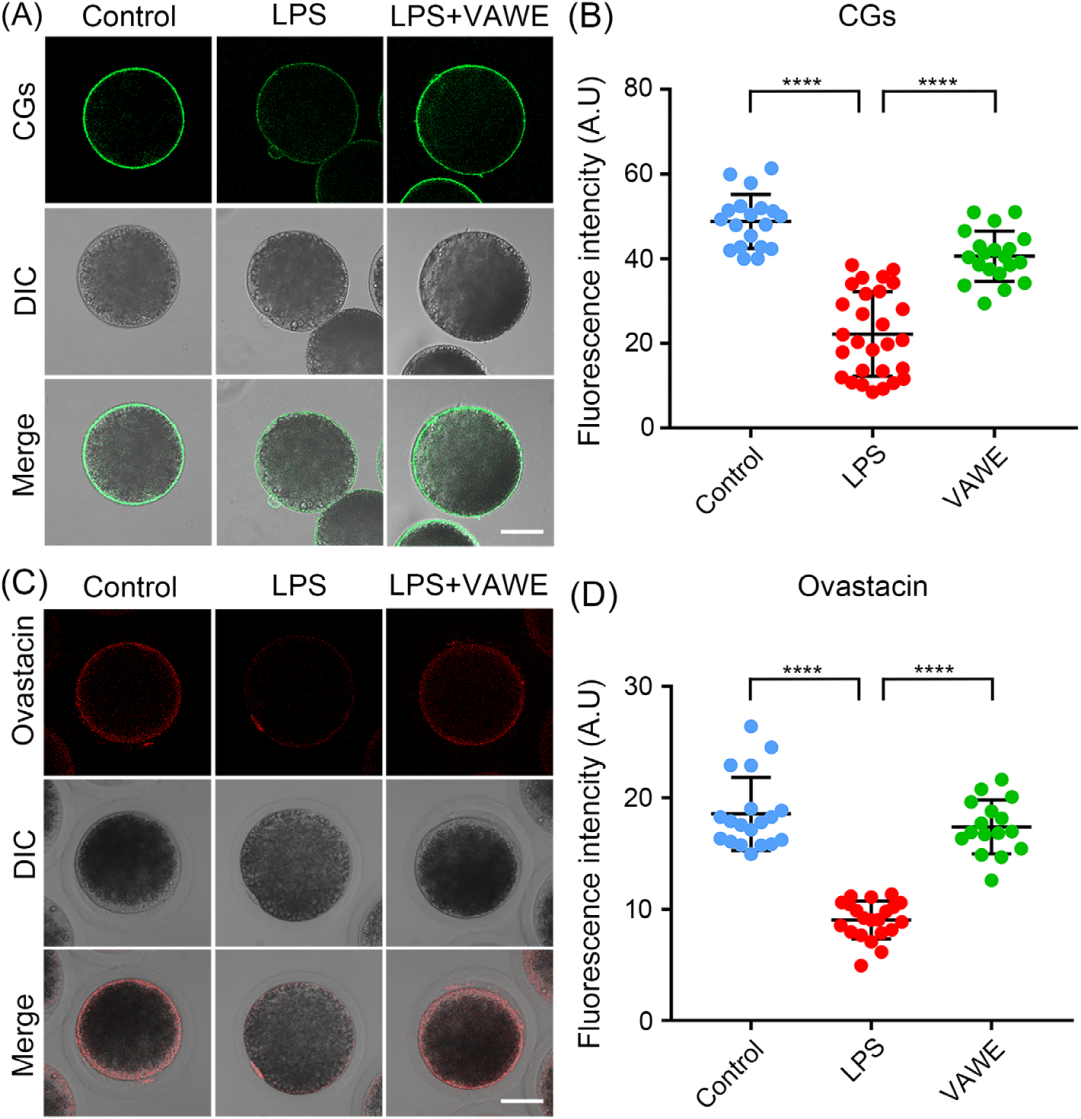
Effects of VAWE supplementation on the localization of CGs and ovastacin in LPS‐exposed porcine oocytes. (A) Representative images of CG distribution in control, LPS‐exposed and VAWE‐supplemented oocytes. Scale bar, 40 μm. (B) The fluorescence intensity of CGs was quantified in control, LPS‐exposed and VAWE‐supplemented oocytes. (C) Representative images of ovastacin distribution in control, LPS‐exposed and VAWE‐supplemented oocytes. Scale bar, 40 μm. (D) The fluorescence intensity of ovastacin signals was measured in control, LPS‐exposed and VAWE‐supplemented oocytes. Data in (B) and (D) were expressed as mean values (mean ± SD) of at least three independent experiments. ****P < 0.0001

As another index of oocyte cytoplasmic maturation, the dynamics of cortical granules (CGs), a particular group of membrane‐bound secretory vesicles that mainly exist in the cortex of mammalian oocytes to function for prevention of polyspermy,^25^ was assessed by LCA‐FITC staining. Under the subcortex of oocytes, we found that LPS exposure perturbed the normal localization of CGs, and signals of CGs were significantly reduced in comparison with the controls (48.8 ± 1.5%, *n* = 19 VS 22.2 ± 1.9%, *n* = 28, *P* < 0.0001; Figure 5A,B). Inversely, treatment with VAWE restored the CG dynamics in LPS‐exposed oocytes (22.2 ± 1.9%, *n* = 28 VS 40.6 ± 1.3%, *n* = 20, *P* < 0.0001; Figure 5A,B).

Besides, we monitored the behaviour of ovastacin, one key component of CGs in LPS‐exposed oocytes. Fluorescent imaging and intensity measurement analysis revealed that the dynamics of ovastacin was similar to that of CGs, showing the lower intensity of ovastacin signals in LPS‐exposed oocytes than those in the controls (18.6 ± 0.8%, *n* = 19 VS 9.0 ± 0.4%, *n* = 21, *P* < 0.0001; Figure **5**C,D). As expected, VAWE supplementation corrected this defect (9.0 ± 0.4%, *n* = 21 VS 17.4 ± 0.6%, *n* = 16, *P* < 0.0001; Figure 5C,D). Altogether, our data illustrate that VAWE promotes the cytoplasmic maturation of oocytes exposed to LPS.

### 3.6 VAWE improves the sperm binding ability and fertilization capacity in porcine oocytes exposed to LPS

**FIGURE 6:**
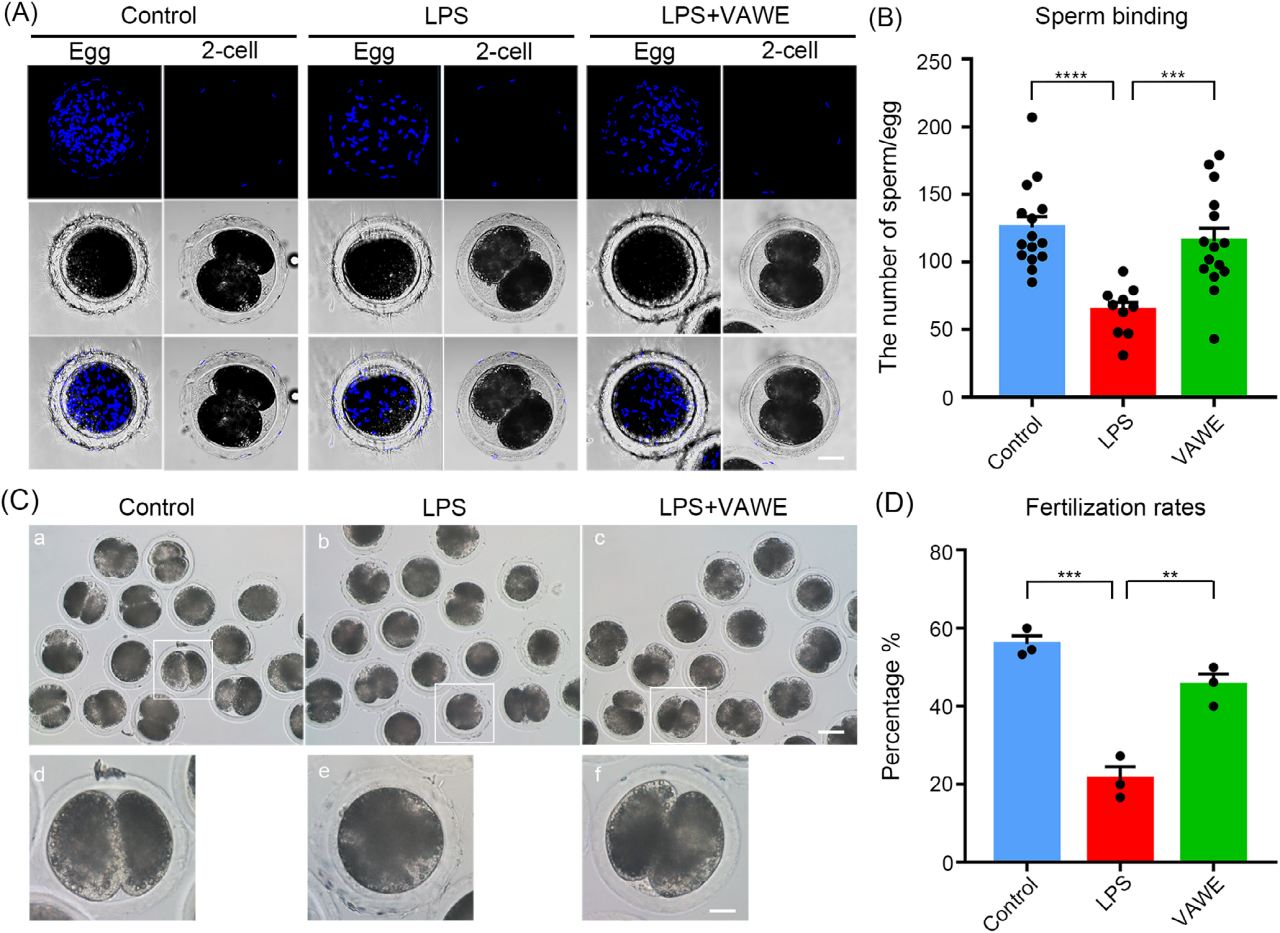
Effects of VAWE supplementation on the sperm binding ability and fertilization potential of LPS‐exposed porcine oocytes. (A) Representative images of sperm binding to the matured oocytes or 2‐cell embryos in control, LPS‐exposed and VAWE‐supplemented groups. Scale bar, 40 μm. (B) The number of sperm binding to the surface of zona pellucida surrounding control, LPS‐exposed and VAWE‐supplemented oocytes was counted. (C) Representative images of 2‐cell embryos developed from in vitro fertilized control, LPS‐exposed and VAWE‐supplemented oocytes. Scale bars, 120 μm (A–C); 30 μm (D–F). (D) The in vitro fertilization rate was quantified in control, LPS‐exposed and VAWE‐supplemented oocytes. Data in (B) and (D) were shown as mean percentage (mean ± SEM) of at least three independent experiments. **P < 0.01; ***P < 0.001; ****P < 0.0001

When ovastacin is exocytosed from CGs to extracellular space before fertilization, it would harden zona pellucida surrounding the oocyte, bringing about unsuccessful sperm binding and fertilization. As the dynamics of ovastacin is impaired in LPS‐exposed oocytes, we performed sperm‐oocyte binging assay to evaluate their sperm binding ability. Sperm heads were stained with Hoechst to count the number of sperm binding to the zona pellucida surrounding oocytes (Figure 6A). The quantitative results displayed that in the control group abundant sperm closely bound to the zona pellucida of unfertilized oocytes, while the number of sperm significantly declined in LPS‐exposed group (125.4 ± 8.2%, *n* = 15 VS 64.3 ± 5.7%, *n* = 10, *P* < 0.0001; Figure 6A,B). By contrast, supplementation of VAWE increased the number of sperm binding to the LPS‐exposed oocytes (64.3 ± 5.7%, *n* = 10 VS 115.3 ± 9.6%, *n* = 15, *P* < 0.0001; Figure 6A,B).

Since weakened sperm binding ability predicts the reduced fertilization potential, we next conducted IVF experiments to confirm it. As shown in Figure 6C,D (55.96 ± 2.05%, *n* = 45 VS 21.31 ± 3.13%, *n* = 45, *P* < 0.001; 21.31 ± 3.13%, *n* = 45 VS 45.38 ± 2.912%, *n* = 45, *P* < 0.01; Figure 6C,D), we observed that LPS‐exposed oocytes developed to fewer 2‐cell embryos than the control oocytes after IVF, whereas VAWE supplementation substantially elevated the IVF rate, suggesting that VAWE enhances the fertilization capacity of LPS‐exposed oocytes by maintaining the sperm binding ability.

### 3.7 VAWE decreases ROS levels to suppress DNA damage and early apoptosis in porcine oocytes exposed to LPS

**FIGURE 7:**
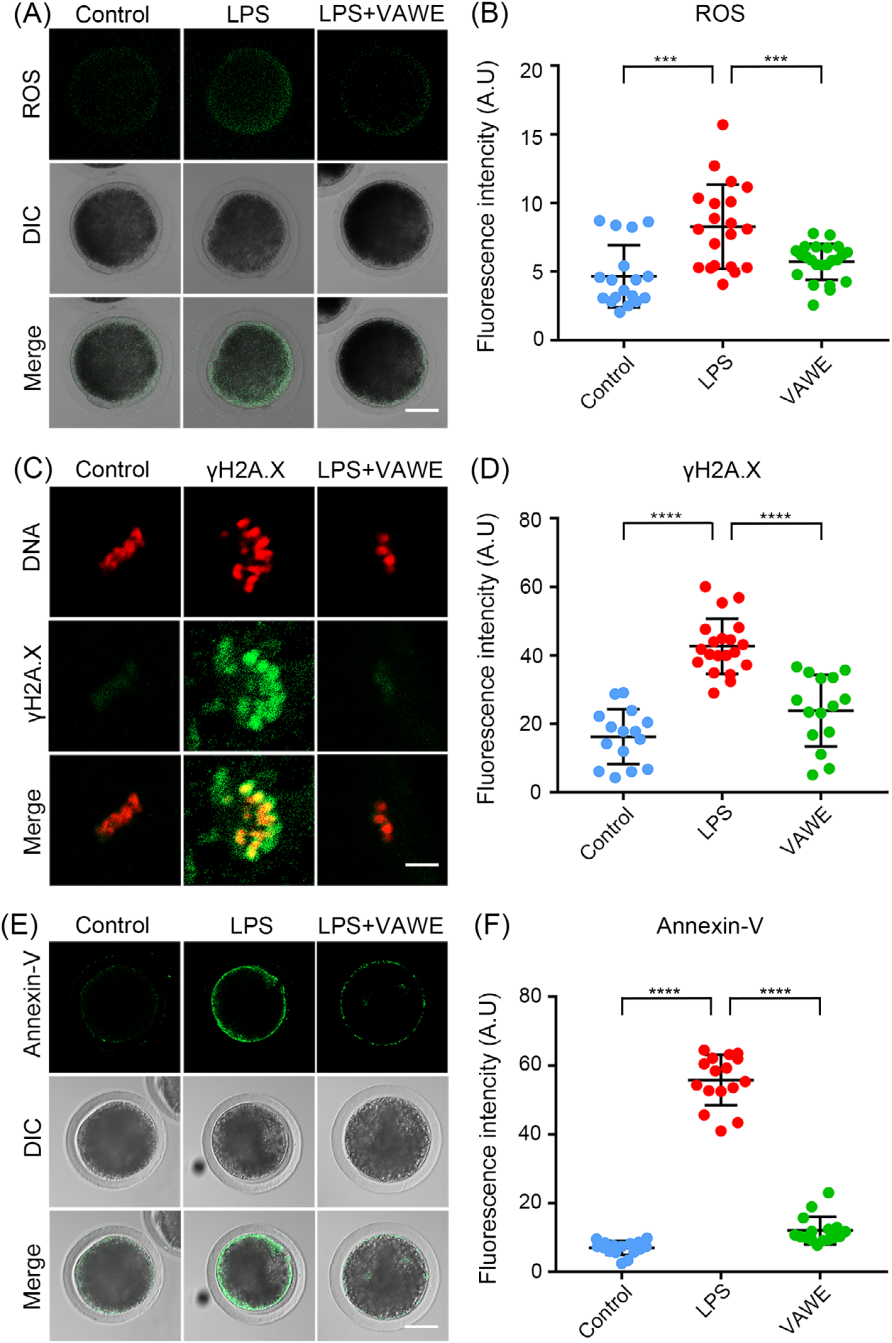
Effects of VAWE supplementation on the ROS level, DNA damage accumulation and apoptosis in LPS‐exposed porcine oocytes. (A) Representative images of ROS levels were shown in control, LPS‐exposed and VAWE‐supplemented oocytes. Scale bar, 40 μm. (B) The fluorescence intensity of ROS signals was measured in control, LPS‐exposed and VAWE‐supplemented oocytes. (C) Representative images of DNA damage were shown in control, LPS‐exposed and VAWE‐supplemented oocytes. Scale bar, 5 μm. (D) The fluorescence intensity of γH2AX signals was measured in control, LPS‐exposed and VAWE‐supplemented oocytes. (E) Representative images of apoptotic oocytes were shown in control, LPS‐exposed and VAWE‐supplemented groups. Scale bar, 40 μm. (F) The fluorescence intensity of Annexin‐V signals was measured in control, LPS‐exposed and VAWE‐supplemented oocytes. Data in (B), (D) and (F) were presented as mean values (mean ± SD) of at least three independent experiments. ***P < 0.001; ****P < 0.0001

It has been reported that LPS‐induced mitochondrial dysfunction produces high levels of reactive oxygen species (ROS) in a variety of cells to impair their functions.^26^, ^27^, ^28^, ^29^ In view of this, we asked whether the improvement of LPS‐exposed oocyte quality by VAWE is related to the removal of ROS. By DCFH staining, ROS levels were compared between control and LPS‐exposed oocytes. The results showed that ROS signals were weak in control oocytes, but became stronger following LPS exposure (4.7 ± 0.5%, *n* = 18 VS 8.3 ± 0.7%, *n* = 20, *P* < 0.001; Figure 7A,B). Nevertheless, supplementation of VAWE sharply reduced the excessive ROS in LPS‐exposed oocytes (8.3 ± 0.7%, *n* = 20 VS 5.7 ± 0.3%, *n* = 23, *P* < 0.001; Figure 7A,B), indicating that VAWE effectively inhibits LPS‐induced oxidative stress in porcine oocytes.

Excessive ROS usually causes the DNA damage accumulation and generates the apoptotic cells, we then tested occurrence of DNA damage and apoptosis in LPS‐exposed oocytes by γH2AX antibody staining and Annexin‐V staining (Figure 7C,E). Quantitative analysis of the fluorescence intensity indicated that LPS exposure significantly increased the signals of γ‐H2AX and Annexin‐V in oocytes (γ‐H2AX: 16.2 ± 2.1%, *n* = 15 VS 42.6 ± 1.8%, *n* = 20, *P* < 0.0001; Annexin‐V: 7.0 ± 0.5%, *n* = 16 VS 55.7 ± 1.8%, *n* = 16, *P* < 0.0001; Figure 7D,F). On the contrary, VAWE supplementation notably reduced these signals, suggesting a lower incidence of DNA damage and apoptosis (γ‐H2AX: 42.6 ± 1.8%, *n* = 20 VS 23.8 ± 2.7%, *n* = 15, *P* < 0.0001; Annexin‐V: 55.7 ± 1.8%, *n* = 16 VS 12.1 ± 1.0%, *n* = 16, *P* < 0.0001; Figure 7D,F).

## 4 DISCUSSION

Velvet antler has a wide range of pharmacological effects which are implicated in promoting the reproductive system development, enhancing immune function, boosting the cell proliferation, and intensifying the anti‐inflammatory and analgesic effects.^12^, ^20^, ^30^ In a previous study, VAWE has been found to improve wound healing in a streptozotocin‐induced diabetic rat model.^12^ In addition, VAWE recovers the mucosal barrier function of colitis,^21^ and protects against cell infection by down‐regulation of pro‐inflammatory cytokines (TNF‐α and IL‐6) and reduction of phagocytosis.^31^, ^32^ Of note, LPS is known as to be responsible for most biological attributes of bacterial endotoxins, which are diffusely present in the environment. LPS induces the release of inflammatory molecules and severely impairs the reproductive health of female animals.^33^, ^34^ Therefore, we hypothesized that VAWE has a protective role against the oxidative damage induced by LPS exposure.^26^, ^27^

Our findings revealed that LPS exposure impeded the porcine oocyte maturation in vitro by impairing the first polar body extrusion and cumulus cell expansion, two key indicators for evaluating the oocyte meiotic progression. Meanwhile, LPS exposure compromised the cytoskeleton organization including meiotic spindle assembly and actin polymerization during oocyte maturation. This might be the major cause leading to the LPS‐induced defects in the oocyte nuclear maturation. Notably we validated that VAME supplementation effectively promoted the in vitro nuclear maturation of LPS‐induced oocytes by recovering the spindle/chromosome structure as well as the actin dynamics.

In addition to the nuclear maturation for ensuring the genomic stability, oocytes need to complete the cytoplasmic maturation to acquire the fertilization ability and subsequent embryonic development potential. The distribution patterns mitochondria and CGs are two well‐accepted indexes for assessing the oocyte cytoplasmic maturation.^35^ Our data demonstrated that VAME supplementation recovered the cytoplasmic maturation of LPS‐exposed oocytes by maintaining the normal distribution and function of mitochondria in oocytes, which might be critical for the generation of energy for oocyte development.^36^, ^37^ Furthermore, we evidenced that VAME supplementation also advanced the cytoplasmic maturation of LPS‐exposed oocytes by restoring the dynamics of CGs and their component ovastacin. As a component of CGs, ovastacin could destroy the sperm binding site in the zona pellucida of oocytes by cleaving the N‐terminal domain of ZP2 to prevent post‐fertilization polyspermy.^38^, ^39^ However, if it is released before fertilization, it would prematurely cleave the ZP2 to impair the sperm binding and fertilization. Consistently, we verified that VAME supplementation enhanced the sperm binding ability and fertilization capability of LPS‐exposed oocytes.

As mitochondrial dysfunction is usually associated with perturbed redox homeostasis, our findings finally illustrated that VAME supplementation reduced the LPS‐induced high levels of ROS and DNA damage in porcine oocytes, and thus suppressing the occurrence of apoptosis.

To sum up, we provide a body of evidence showing that VAWE protects oocyte quality from LPS‐induced deterioration by promoting both nuclear and cytoplasmic maturation of oocyte meiosis via removal of excessive ROS and inhibition of apoptosis.

## AUTHOR CONTRIBUTIONS

Baozeng Xu designed the research; Jingyue Chen, Rui Wang, Yilong Miao, C. Rao, Qian Gao performed experiment; Chunxiao Liu, Huimin Sun provided the materials; Jingyue Chen, Bo Xiong, Baozeng Xu analysed the data; Jingyue Chen, Baozeng Xu wrote original draft; Bo Xiong edited the draft.

## CONFLICT OF INTEREST

The authors declare that they have no conflict of interest.

## Supporting information


**Table S1.**Supplemental table.

## Data Availability

The data used to support the findings of this study are available from the corresponding author upon request.

## References

[1] Adetunji AO , Kawai T , Shimada M . Impact of lipopolysaccharide administration on luteinizing hormone/choriogonadotropin receptor (Lhcgr) expression in mouse ovaries. J Reprod Immunol. 2020;142:103193. 32890905 10.1016/j.jri.2020.103193

[2] Lundin JI , Checkoway H . Endotoxin and cancer. Environ Health Perspect. 2009;117(9):1344‐1350. 19750096 10.1289/ehp.0800439 PMC2737008

[3] Forrest KK , Flores VV , Gurule SC , et al. Effects of lipopolysaccharide on follicular estrogen production and developmental competence in bovine oocytes. Anim Reprod Sci. 2022;237:106927. 35074697 10.1016/j.anireprosci.2022.106927 PMC8928215

[4] Rasekhi M , Mohammadi‐Sangcheshmeh A , Daliri M , et al. Transcriptional profile of ovine oocytes matured under lipopolysaccharide treatment in vitro. Theriogenology. 2020;157:70‐78. 32805644 10.1016/j.theriogenology.2020.07.034

[5] Tuo W , Ott TL , Liu S , Bazer FW . Intrauterine infusion of bacterial lipopolysaccharide (LPS) prior to mating has no adverse effect on fertility, fetal survival and fetal development. J Reprod Immunol. 1999;42(1):31‐39. 10098830 10.1016/s0165-0378(98)00078-3

[6] Bromfield JJ , Sheldon IM . Lipopolysaccharide initiates inflammation in bovine granulosa cells via the TLR4 pathway and perturbs oocyte meiotic progression in vitro. Endocrinology. 2011;152(12):5029‐5040. 21990308 10.1210/en.2011-1124 PMC3428914

[7] Bidne KL , Dickson MJ , Ross JW , Baumgard LH , Keating AF . Disruption of female reproductive function by endotoxins. Reproduction. 2018;155(4):R169‐R181. 29363567 10.1530/REP-17-0406

[8] Yan K , Cui K , Nie J , et al. Mogroside V protects porcine oocytes from lipopolysaccharide‐induced meiotic defects. Front Cell Dev Biol. 2021;9:639691. 33763421 10.3389/fcell.2021.639691 PMC7982822

[9] Li Q , Zhang Y , Li W , et al. Allicin protects porcine oocytes against LPS‐induced defects during maturation in vitro. Theriogenology. 2022;182:138‐147. 35176679 10.1016/j.theriogenology.2022.02.007

[10] Magata F , Shimizu T . Effect of lipopolysaccharide on developmental competence of oocytes. Reprod Toxicol. 2017;71:1‐7. 28408308 10.1016/j.reprotox.2017.04.001

[11] Wang X , Li H , Liu Y , et al. Velvet antler methanol extracts (MEs) protects against oxidative stress in Caenorhabditis elegans by SKN‐1. Biomed Pharmacother. 2020;121:109668. 31766103 10.1016/j.biopha.2019.109668

[12] Mikler JR , Theoret CL , High JC . Effects of topical elk velvet antler on cutaneous wound healing in streptozotocin‐induced diabetic rats. J Altern Complement Med. 2004;10(5):835‐840. 15650473 10.1089/acm.2004.10.835

[13] Jiang C , Jin Y , Zhao X , Yuan Y , Zhao Y , Huang L . Rapid and robust authentication of deer antler velvet product by fast PCR‐RFLP analysis. Mitochondrial DNA A DNA Mapp Seq Anal. 2018;29(2):266‐272. 28071968 10.1080/24701394.2016.1275599

[14] Tseng SH , Sung CH , Chen LG , et al. Comparison of chemical compositions and osteoprotective effects of different sections of velvet antler. J Ethnopharmacol. 2014;151(1):352‐360. 24212078 10.1016/j.jep.2013.10.060

[15] Xiao X , Li L , Xu S , et al. Evaluation of velvet antler total protein effect on bone marrow‐derived endothelial progenitor cells. Mol Med Rep. 2017;16(3):3161‐3168. 28714033 10.3892/mmr.2017.7019 PMC5547914

[16] Xiao X , Xu S , Li L , et al. The effect of velvet antler proteins on cardiac microvascular endothelial cells challenged with ischemia‐hypoxia. Front Pharmacol. 2017;8:601. 28936174 10.3389/fphar.2017.00601 PMC5595159

[17] Cheng WJ , Yang HT , Chiang CC , et al. Deer velvet antler extracts exert anti‐inflammatory and anti‐arthritic effects on human rheumatoid arthritis fibroblast‐like synoviocytes and distinct mouse arthritis. Am J Chin Med. 2022;50(6):1617‐1643. 35850642 10.1142/S0192415X22500689

[18] Liu Y , Li H , Li Y , Yang M , Wang X , Peng Y . Velvet antler methanol extracts ameliorate Parkinson's disease by inhibiting oxidative stress and neuroinflammation: from *C. elegans* to mice. Oxid Med Cell Longev. 2021;2021:8864395. 33505591 10.1155/2021/8864395 PMC7811427

[19] Kim HS , Lim HK . Inhibitory effects of velvet antler water extract on morphine‐induced conditioned place preference and DA receptor supersensitivity in mice. J Ethnopharmacol. 1999;66(1):25‐31. 10432204 10.1016/s0378-8741(98)00195-0

[20] Kim HS , Lim HK , Park WK . Antinarcotic effects of the velvet antler water extract on morphine in mice. J Ethnopharmacol. 1999;66(1):41‐49. 10432206 10.1016/s0378-8741(98)00193-7

[21] Hung YK , Ho ST , Kuo CY , Chen MJ . In vitro effects of velvet antler water extracts from Formosan Sambar deer and red deer on barrier integrity in Caco‐2 cell. Int J Med Sci. 2021;18(8):1778‐1785. 33746595 10.7150/ijms.53599 PMC7976581

[22] Chang JS , Lin HJ , Deng JS , Wu WT , Huang SS , Huang GJ . Preventive effects of velvet antler (*Cervus elaphus*) against lipopolysaccharide‐induced acute lung injury in mice by inhibiting MAPK/NF‐kappaB activation and inducing AMPK/Nrf2 pathways. Evid Based Complement Alternat Med. 2018;2018:2870503. 29483931 10.1155/2018/2870503 PMC5816838

[23] Du F , Zhao H , Yao M , Yang Y , Jiao J , Li C . Deer antler extracts reduce amyloid‐beta toxicity in a *Caenorhabditis elegans* model of Alzheimer's disease. J Ethnopharmacol. 2022;285:114850. 34801608 10.1016/j.jep.2021.114850

[24] Shi H , Zhao T , Li Y , et al. Velvet antler ameliorates cardiac function by restoring sarcoplasmic reticulum Ca(2+)‐ATPase activity in rats with heart failure after myocardial infarction. Front Pharmacol. 2021;12:621194. 33995020 10.3389/fphar.2021.621194 PMC8120434

[25] Wang WH , Sun QY , Hosoe M , Shioya Y , Day BN . Quantified analysis of cortical granule distribution and exocytosis of porcine oocytes during meiotic maturation and activation. Biol Reprod. 1997;56(6):1376‐1382. 9166688 10.1095/biolreprod56.6.1376

[26] Kawai K , Yamamoto M , Kameyama S , Kawamata H , Rademaker A , Oyasu R . Enhancement of rat urinary bladder tumorigenesis by lipopolysaccharide‐induced inflammation. Cancer Res. 1993;53(21):5172‐5175. 8221653

[27] Sprong RC , Aarsman CJ , van Oirschot JF , van Asbeck BS . Dimethylthiourea protects rats against gram‐negative sepsis and decreases tumor necrosis factor and nuclear factor kappaB activity. J Lab Clin Med. 1997;129(4):470‐481. 9104891 10.1016/s0022-2143(97)90081-0

[28] Spolarics Z . Endotoxemia, pentose cycle, and the oxidant/antioxidant balance in the hepatic sinusoid. J Leukoc Biol. 1998;63(5):534‐541. 9581796 10.1002/jlb.63.5.534

[29] Loft S , Deng XS , Tuo J , Wellejus A , Sorensen M , Poulsen HE . Experimental study of oxidative DNA damage. Free Radic Res. 1998;29(6):525‐539. 10098457 10.1080/10715769800300571

[30] Kuo CY , Cheng YT , Ho ST , Yu CC , Chen MJ . Comparison of anti‐inflammatory effect and protein profile between the water extracts from Formosan sambar deer and red deer. J Food Drug Anal. 2018;26(4):1275‐1282. 30249326 10.1016/j.jfda.2018.02.005 PMC9298571

[31] Kuo CY , Wang T , Dai TY , et al. Effect of the velvet antler of Formosan sambar deer (*Cervus unicolor swinhoei*) on the prevention of an allergic airway response in mice. Evid Based Complement Alternat Med. 2012;2012:481318. 23346203 10.1155/2012/481318 PMC3544540

[32] Dai TY , Wang CH , Chen KN , et al. The antiinfective effects of velvet antler of Formosan sambar deer (*Cervus unicolor swinhoei*) on *Staphylococcus aureus*‐infected mice. Evid Based Complement Alternat Med. 2011;2011:534069. 21584242 10.1155/2011/534069 PMC3092581

[33] Carvalho FB , Gutierres JM , Bueno A , et al. Anthocyanins control neuroinflammation and consequent memory dysfunction in mice exposed to lipopolysaccharide. Mol Neurobiol. 2017;54(5):3350‐3367. 27167130 10.1007/s12035-016-9900-8

[34] Shakil T , Snell A , Whitehead SA . Effects of lipopolysaccharide and cyclosporin on the endocrine control of ovarian function. J Reprod Fertil. 1994;100(1):57‐64. 8182612 10.1530/jrf.0.1000057

[35] Miao Y , Cui Z , Gao Q , Rui R , Xiong B . Nicotinamide mononucleotide supplementation reverses the declining quality of maternally aged oocytes. Cell Rep. 2020;32(5):107987. 32755581 10.1016/j.celrep.2020.107987

[36] Zhou C , Zhang X , Chen Y , Liu X , Sun Y , Xiong B . Glutathione alleviates the cadmium exposure‐caused porcine oocyte meiotic defects via eliminating the excessive ROS. Environ Pollut. 2019;255(Pt 1):113194. 31520902 10.1016/j.envpol.2019.113194

[37] Dai X , Qiu L , Zhao B , et al. Melatonin ameliorates the fertilization capacity of oocytes exposed to 17alpha‐ethynylestradiol. Reprod Toxicol. 2020;93:61‐67. 31931096 10.1016/j.reprotox.2020.01.004

[38] Chen J , Miao Y , Gao Q , Cui Z , Xiong B . Exposure to perfluorooctane sulfonate in vitro perturbs the quality of porcine oocytes via induction of apoptosis. Environ Pollut. 2021;284:117508. 34261219 10.1016/j.envpol.2021.117508

[39] Zhang M , Miao Y , Chen Q , et al. BaP exposure causes oocyte meiotic arrest and fertilization failure to weaken female fertility. FASEB J. 2018;32(1):342‐352. 28904021 10.1096/fj.201700514R

